# Impact of Bariatric Surgery on Renal Function

**DOI:** 10.7759/cureus.18534

**Published:** 2021-10-06

**Authors:** Dickson Dewantoro, Joshua Fultang, Katie Lowe, Ugochukwu Chinaka, Andisheh Bakhshi, Abdulmajid Ali

**Affiliations:** 1 Bariatric Surgery, University Hospital Ayr, Ayr, GBR; 2 Surgery, University of Glasgow, Glasgow, GBR; 3 Bariatric Surgery, University Hospital Ayr/University of the West of Scotland, Ayr, GBR; 4 General Surgery, University Hospital Ayr/University of the West of Scotland, Ayr, GBR; 5 School of Computing, Engineering and Physical Sciences (CEPS), University of the West of Scotland, Paisley, GBR; 6 General and Upper Gastrointestinal Surgery/Bariatric & Metabolic Surgery, University Hospital Ayr, Ayr, GBR; 7 School of Health and Science, University of the West of Scotland, Paisley, GBR

**Keywords:** obesity, effect of bariatric surgery, post operative, renal function, bariatric surgery

## Abstract

Introduction

Bariatric surgery offers superior benefits for weight loss, quality of life and a spectrum of metabolic diseases. Despite these benefits, studies so far have shown varying results on its effect on renal function.

Aim

In this study, we aim to look at bariatric surgery’s effect on renal function at one, two and three year post operation (post-op).

Methods

This is a retrospective cross-sectional single-center study of patients who underwent bariatric surgery between 11/2008 and 06/2018. Renal function was calculated by using Cockroft-Gault equation, expressed as Creatinine Clearance (CrCl). Statistical analysis used was one-way ANOVA (Welch’s) with Games-Howell Post-Hoc Test.

Results

From 307 patients who underwent bariatric surgery within the time period, 145 were studied. 30.3% (n=44) were male. The average age and body mass index (BMI) at referral were 48.1±8.6 years and 47.96±7.9 kgm^-2^ respectively, while the average age and BMI at surgery were 49.1±8.8 years and 40.62±4.2 kgm^-2^ respectively. Mean CrCl at surgery, year 1, year 2, and year 3 post-op were 172.35±53.29 mL/min, 179.20±57.87 mL/min, 142.35±46.05 mL/min, and 119.56±42.46 mL/min. Marginal improvement of CrCl at year one post-op (172.35±53.29mL/min to 179.20±57.87mL/min) was statistically insignificant (p=0.731). Meanwhile, there was statistically significant CrCl decline observed from year 1 to year 3 post-op (p<0.001).

Conclusion

Statistically insignificant marginal improvement in CrCl at year one post-op was noted. Beyond this, there was steady CrCl decline, albeit remained higher than the lower limit for respective gender. We recommend for further studies that take into account additional factors affecting renal function.

## Introduction

Bariatric surgery offers superior mid to long-term outcomes in terms of weight loss, quality of life and a spectrum of metabolic diseases including type II diabetes [1,2]. Bariatric surgery which involves surgical manipulation of the gastro-intestinal tract with achieving weight loss, as a prime objective, can be grouped into restrictive, malabsorptive and combined procedures. Example of these procedures include gastric banding, sleeve gastrectomy, distal gastric or jejunoileal bypass, biliopancreatic diversion, duodenal switch; and Roux-en-Y gastric bypass, respectively [3].

Some have opined that together with these metabolic improvements, patients experience an improvement in renal function [1,2,4]. Others suggest bariatric surgery has a neutral effect on renal function [1]. More alarmingly, some controversial evidence has emerged to suggest a deterioration in renal function following bariatric surgery [5].

In this study, we explore the impact of bariatric surgery (Gastric sleeve (SG) and Roux-en-Y bypass (RYBG)) on renal function in the short to midterm postoperative period. This was achieved by analysing the creatinine clearance of patients who underwent bariatric surgery at one, two and three years postoperatively.

## Materials and methods

This was a retrospective cross-sectional single-center study performed by analysing data from November 2008 to June 2018. 307 patients underwent bariatric surgery (SG or RYGB) during the timeframe of the study. Of these, data to calculate creatinine clearance (CrCl) for 145 patients was available. Results were obtained by searching through the bariatric database and clinical portal. These results included Body Mass Index (BMI), weight, gender, serum creatinine, height and co-morbidities preoperatively and at one-, two- and three-year postoperatively. The remaining 162 patients who underwent bariatric surgery were excluded as there were no renal function blood tests done at the respective year of interest.

**Figure 1:**
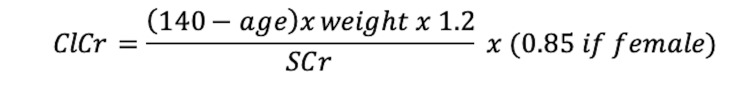
Cockroft-Gault equation. Where ClCr is creatinine clearance; SCr is serum creatinine in µmol/L; and weight in kg [6,7].

The cockroft-Gault equation was used to calculate creatinine clearance at the interval of interest. Cockroft-Gault Equation as defined in by D W Cockroft and M H Gault in 1976 [6,7]. This formula is shown in Figure 1. Appropriate value for age, weight, and serum creatinine were collected at the timeframe of interest. Analysis was performed using Microsoft Excel 2016.

Data analysis

One-way ANOVA (Welch’s) calculation with a Games-Howell Post-Hoc Test was done in order to assess the significance of the difference in Creatinine Clearance between time points. A 5% significance critical level for creatinine clearance was adopted.

Exclusion criteria

Patients with insufficient data defined as lacking the variable of interest- operatively, one, two and three year post operatively were excluded. This resulted in the reduction of patient number from 145 at time of surgery, to 137; 119; and 93, at year 1; 2; and 3 following surgery, respectively.

## Results

Data for 145 patients included in this study, 44 were male (30.3%). The average age at referral was 48.1±8.6 years while the average age at surgery was 49.1±8.8 years. More patients in the study underwent SG 57.9% (n=84) and 61 patients underwent RYGB (42.1%). Of all these procedures, 13 patients (9%) underwent revision surgery (conversion from a gastric band). 98 patients had a diagnosis of type II diabetes (T2DM) of which six were diet controlled (6.1%) while the rest were on pharmacological treatment with oral antidiabetic medication with or without insulin therapy. The average BMI at the time of referral was 47.96±7.9 kgm-2 while average BMI at surgery was 40.62±4.2 kgm-2 reflecting a net mandatory pre-operation weight loss.

As seen in Table 1, when comparing the mean creatinine clearance (CrCl) at surgery was 172.35±53.29 mL/minute. When comparing the mean value of CrCl at the time of surgery to, year 1, year 2, and year 3 following surgery, the mean value of CrCl were 172.35±53.29 mL/min, 179.20±57.87 mL/min, 142.35±46.05 mL/min, and 119.56±42.46 mL/min, respectively. 

**Table 1:** Mean creatinine clearance (CrCl) at surgery and postoperatively.

Creatinine clearance	Time	N	Mean	SD	SE
	Surgery	145	172.35	53.29	4.43
	Year 1	137	179.2	57.87	4.94
	Year 2	119	142.35	46.05	4.22
	Year 3	93	119.56	42.46	4.4

**Figure 2:**
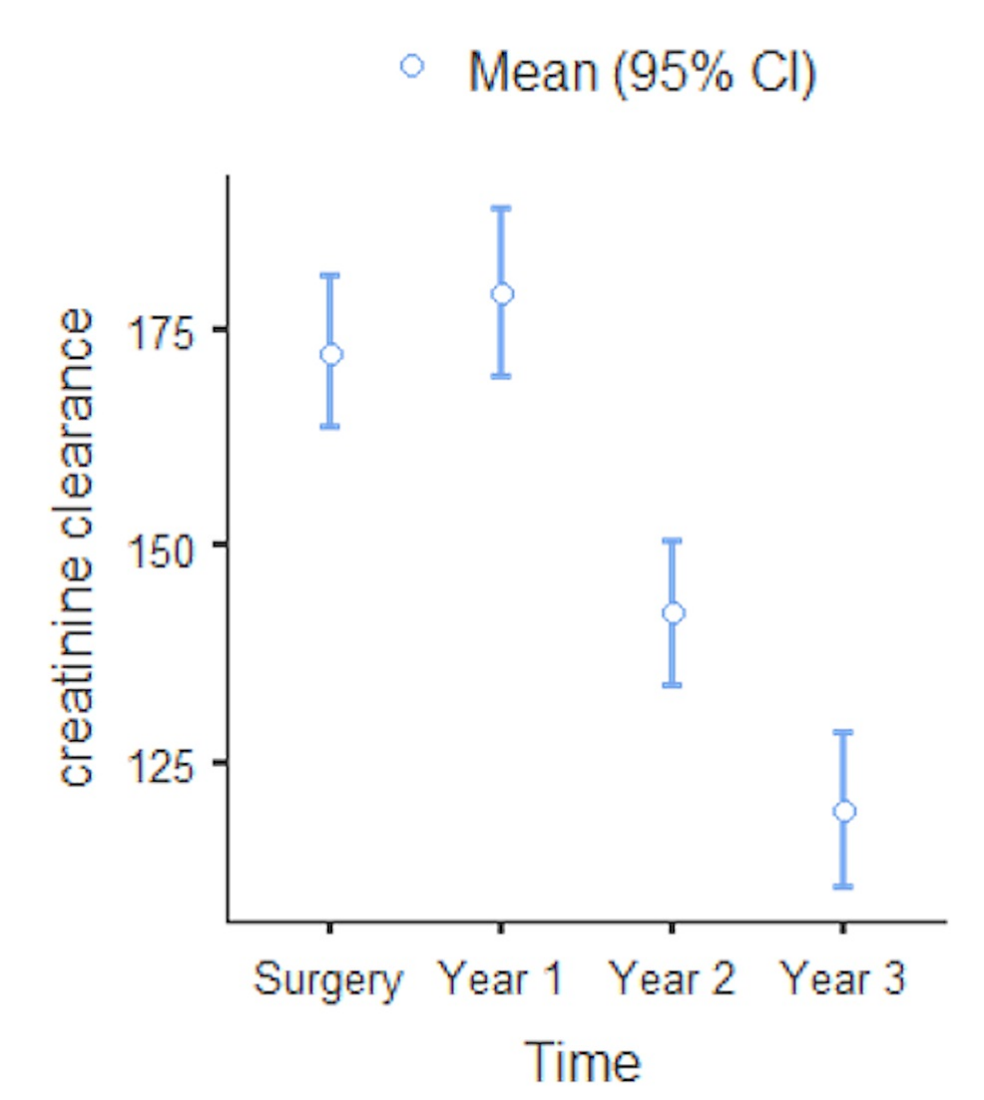
Mean creatinine clearance versus postoperative years with 95% confidence interval.

As seen in Figure 2, there was a marginal improvement in CrCl at year 1 post-operatively (from 172.35±53.29 mL/min to 179.20±57.87 mL/min). However as seen from Table 2, this improvement was not statistically significant (p=0.731).

Interestingly, there was a decline in CrCl in the short- to mid-period where CrCl dropped progressively from 1 to 2 and 3 years postoperatively. This decline was statistically significant at every interval as seen in Table 2. Clinically, this decline in CrCl was still above the lower limit of normal of 110 mL/min in males and 100 mL/min in females. 

**Table 2:** Showing Games-Howell post-hoc test – CrCl mL/min. *p < 0.05, **p < 0.01, ***p < 0.001. CrCl: creatinine clearance.

		Surgery	Year 1	Year 2	Year 3
Surgery	Mean difference	—	-6.85	30.01***	52.80***
	t-value	—	-1.03	4.91	8.46
	df	—	274.69	261.28	225.04
	p-value	—	0.731		
Year 1	Mean difference		—	36.86***	59.65***
	t-value		—	5.67	9.01
	df		—	252.13	226.59
	p-value		—		
Year 2	Mean difference			—	22.79**
	t-value			—	3.74
	df			—	204.3
	p-value			—	0.001

As seen from above, there was a statistically significant difference in CrCl from 1 to 2 years post-operatively and 2 to 3 years post-operatively (p<0.001).

## Discussion

Renal function can be assessed by various methods. In this study, creatinine clearance was used as it provides an evidence-based, cost-effective and minimally invasive assessment of renal function. Creatinine is a product of creatine and phosphocreatine metabolism, found in skeletal muscle, freely filtered by glomerulus and not reabsorbed [8,9]. This makes creatinine to be an ideal endogenous substance for glomerular filtration rate measurement [10].

CrCl can be measured by collecting 24 hours urine and measuring the amount of creatinine in a given volume of urine. This value would then be divided by the value of plasma creatinine to provide CrCl. This method of measuring CrCl, which correlates to renal function, is time-consuming. There are several formulas that can be used to estimate CrCl, one of which is Cockroft-Gault formula as previously discussed [11].

Our study showed there was a statistically insignificant marginal improvement in CrCl at 1 year postoperatively. This finding is similar to that of a retrospective study published in 2018, which compared laparoscopic Roux-en-Y gastric bypass and laparoscopic sleeve gastrectomy. The study showed improvement of renal function albeit being almost two years following surgery [12]. 

However, beyond this (2 and 3 years postoperatively) there was a steady decline in creatinine clearance. This is supported by a case report study that showed renal failure is a recognised complication of bariatric surgery, especially when techniques used are Roux-en-Y gastric bypass or jejunoileal bypass. The main cause of renal failure in mentioned study being Acute, lead by diarrhoea+/- vomiting or intratubular calcium oxalate deposition, which is likely to be reversible given careful monitoring [5].

Despite these declines in CrCl, the average CrCl remained higher the lower limit of normal for males and females. The normal value for male is 110-150 mL/min while the normal value for females is 100-130 mL/min [13]. The reasons for this decline in CrCl remain uncertain and may be multifactorial. As discussed previously, in Cockroft-Gault equation, various physiological aspects of patient (most importantly in this case, weight and age) are taken into account. Given that patients in the study lie within the extreme ends of obesity (40.62±4.2 kgm-2 ) and all underwent procedures to lose weight these factors reduce the reliability of the Cockroft-Gault equation [10]. 

One limitation to this study centre around the use of creatinine to estimate renal function. Creatinine is an endogenous substance that is able to provide a relatively accurate reflection of renal function, however, there is another substance that can provide a more accurate reflection of renal function calculation, albeit being an exogenous substance and expensive, which is inulin [10]. Inulin can achieve this as it is freely filtered in the glomerulus and is not metabolised, reabsorbed and secreted in the tubules [14].

There are factors that need to be considered while using creatinine as estimation of kidney function (glomerular filtration rate). Serum creatinine is dependent on weight, age, and sex [6]. Cockroft-Gault Equation seen in Figure 1 is one of the formulas that take into account such factors and ensuring a more accurate measurement of creatinine clearance, thus a more accurate measurement of glomerular filtration rate. However, there may be other factors outwith the formula that were responsible to cause the observed CrCl decline. To know this for certain, further studies need to be done in order to study the exact cause of CrCl decline, preferably, one which includes comorbidities and lifestyle.

The retrospective nature of this study and the fact that it is being performed in a single-centre means that there are several limitations to the study. Firstly, the data that was collected was not specifically done with this study in mind. Thus, there are several patients with missing data and this has quite significantly reduced the number of patients being included in the study. Secondly, unknown and unnoticed biases cannot be fully accounted for in this study. Factors such as detailed co-morbidities and lifestyle were not taken into account in this study which may contribute to the observed effect on renal function. Thirdly, there was no control of exposure of patients toward unrecognised confounders that may affect renal function. Lastly, being single-centred study, it may not represent the general population due to the restricted ration of races involved [15,16].

Therefore, further study which will include co-morbidities that may greatly impact on patient’s renal function, within multiple centre, should be done in order to further study the absolute cause of decline of CrCl in post-bariatric surgery patients.

## Conclusions

In this cross-sectional single centre retrospective study, we studied 145 patients who underwent bariatric surgery within NHS Ayrshire and Arran. Their CrCl were calculated at time of surgery, year 1, year 2 and year 3 post-operatively by using Cockroft-Gault Equation. We found that there was a statistically insignificant marginal improvement in CrCl at 1 year postoperatively. However, beyond this (2 and 3 years postoperatively), there was a steady decline in creatinine clearance, albeit the values remained within the reference range of normal CrCl for the respective gender. In this study, we did not take into account other factors that affect renal function and therefore, we recommend for further studies that take into account various factors affecting renal function. 
